# An *APETALA1* ortholog affects plant architecture and seed yield component in oilseed rape (*Brassica napus* L.)

**DOI:** 10.1186/s12870-018-1606-9

**Published:** 2018-12-29

**Authors:** Smit Shah, Nirosha L. Karunarathna, Christian Jung, Nazgol Emrani

**Affiliations:** Plant Breeding Institute, Christian-Albrechts-University of Kiel, Olshausenstr. 40, 24098 Kiel, Germany

**Keywords:** Meristem identity genes, TILLING, EMS-induced mutations, Plant height, Branch height, Seed yield

## Abstract

**Background:**

Increasing the productivity of rapeseed as one of the widely cultivated oil crops in the world is of upmost importance. As flowering time and plant architecture play a key role in the regulation of rapeseed yield, understanding the genetic mechanism underlying these traits can boost the rapeseed breeding. Meristem identity genes are known to have pleiotropic effects on plant architecture and seed yield in various crops. To understand the function of one of the meristem identity genes, *APETALA1* (*AP1*) in rapeseed, we performed phenotypic analysis of TILLING mutants under greenhouse conditions. Three stop codon mutant families carrying a mutation in *Bna.AP1.A02* paralog were analyzed for different plant architecture and seed yield-related traits.

**Results:**

It was evident that stop codon mutation in the K domain of *Bna.AP1.A02* paralog caused significant changes in flower morphology as well as plant architecture related traits like plant height, branch height, and branch number. Furthermore, yield-related traits like seed yield per plant and number of seeds per plants were also significantly altered in the same mutant family. Apart from phenotypic changes, stop codon mutation in K domain of *Bna.AP1.A02* paralog also altered the expression of putative downstream target genes like *Bna.TFL1* and *Bna.FUL* in shoot apical meristem (SAM) of rapeseed. Mutant plants carrying stop codon mutations in the COOH domain of *Bna.AP1.A02* paralog did not have a significant effect on plant architecture, yield-related traits or the expression of the downstream targets.

**Conclusions:**

We found that *Bna.AP1.A02* paralog has pleiotropic effect on plant architecture and yield-related traits in rapeseed. The allele we found in the current study with a beneficial effect on seed yield can be incorporated into rapeseed breeding pool to develop new varieties.

**Electronic supplementary material:**

The online version of this article (10.1186/s12870-018-1606-9) contains supplementary material, which is available to authorized users.

## Background

Rapeseed (*Brassica napus* L., AACC, 2n = 38) is one of the most important oil crops in the world for the production of vegetable oil and animal feed. The productivity of rapeseed has been significantly increased in the last ten years, mainly due to the high yielding cultivars, mechanical harvesting, and better agronomic practices. Nevertheless, to meet the increasing demand for edible oil worldwide, it is important to understand the genetic mechanism underlying rapeseed productivity [1]. To increase the productivity, optimization of flowering time and plant architecture is fundamental.

Flowering time plays a very crucial role in the environmental adaptation of the plant. Environmental adaptation mainly includes the adaptation to prevailing climatic conditions (for example season, day length, and temperature), as well as biotic and abiotic stresses. The adaptation to the environment is important for the overall yield of the plant. It is known from the model plant Arabidopsis that environmental factors like cold temperature, photoperiod, and ambient temperature, as well as, genetic and epigenetic factors, influence floral transition. Under long day conditions, the floral inducers *CONSTANS* (*CO*) and *FLOWERING LOCUS T* (*FT*) are activated and trigger the expression of meristem identity genes like *LEAFY* (*LFY*), *APETALA1* (*AP1*)*, SEPALLATA3* (*SEP3*) and *FRUITFULL* (*FUL*). Subsequently, meristem identity genes transform the Arabidopsis shoot apical meristem into a floral meristem [2]. In contrast to *FT*, *TERMINAL FLOWER-1* (*TFL1*), which shares high sequence similarity (71%) with *FT*, represses downstream meristem identity genes such as *AP1* and *LFY* in the central zone of the meristem. Considering the close phylogenetic relationship between rapeseed and Arabidopsis, knowledge of flowering time control in Arabidopsis provides the basis to understand the regulatory network in rapeseed. However, knowledge transfer from Arabidopsis to rapeseed is hindered by the complexity of the rapeseed genome. Different approaches have been applied to unveil the flowering time mechanism in rapeseed. Application of bi-parental populations for mapping quantitative trait loci (QTL) for flowering time and other agronomic traits is one of the widely used approaches [3–5]. In one of such studies, Raman et al. [6] mapped 20 different flowering time loci to 10 different chromosomes using a doubled haploid (DH) population, derived from a cross between two vernalization responsive rapeseed cultivars. They reported that three paralogs of *Bna.AP1* coincided with flowering time QTL on chromosomes A02, A07, and A08. Nevertheless, up to now no studies in rapeseed have demonstrated the phenotypic effect of *Bna.AP1* overexpression or mutation on flowering time.

In addition to flowering time, selecting plants with ideal architecture is also crucial for crop domestication and improvement [7]. Therefore, over the years, there have been several studies in major crops to understand the mechanisms, which control plant architecture. For example, in rice, the ideal plant architecture (IPA) plant was reported to have thicker and more robust stems with more grains per panicle [7]. Variation in the leaf angle also showed a significant effect on maize grain yield, underpinning the importance of plant architecture in optimizing crop yield [8, 9]. Plant height, branch length, branch angle, length of main inflorescences, leaf angle and branch number per plant define plant architecture in *B. napus*, which affect seed yield components like silique number per plant and also number of seeds per plant [10, 11]. Cai et al. [1] mapped 163 QTL related to plant architecture and yield-related traits in a DH population comprising 254 individuals. In another study, Shen et al. [12] could identify 19 QTL related to plant height, branch initiation height, stem diameter and flowering time in a DH population with 208 individuals. Using the same population, 17 QTL for branch angle were found, of which, three major QTL were steadily expressed, each explaining more than 10% of the phenotypic variation [13]. Besides genetic mapping, association mapping has also been used to find candidate genes for the trait of interest. In one of such GWAS studies [14], 158 winter rapeseed accessions were phenotyped for flowering time, plant height and seed yield in 11 different environments across Germany, China and Chile. These accessions were genotyped using the Brassica 60 K-SNP Illumina® Infinium consortium array. They found 68 regions across the rapeseed genome, which showed multi-trait associations. Using the same SNP array, Li et al. [15] genotyped 472 diverse rapeseed accessions to find regions associated with rapeseed branch angle. They found 21 loci across the genome with significant associations with branch angle. In another GWAS study, Zheng et al. [16] genotyped and phenotyped 333 rapeseed accessions across 4 years and found seven loci for plant height, four for branch initiation height, and five for branch number.

During plant development, the shoot apical meristem (SAM) transforms into an inflorescence meristem and finally into a floral meristem. Subsequently, sepals, petals, stamen, and carpel of the flower are developed from the floral meristem. Several meristem identity genes play an important role in the development of floral organs. Moreover, there are reports in crops suggesting the role of meristem identity genes in controlling plant architecture [17–19]. Meristem identity and determinacy are controlled by the overlapping expression of meristem identity genes of the ABCE model [20]. In Arabidopsis, A-functional genes include *APETALA1*(*AP1*) and *APETALA2*(*AP2*), B- functional genes include *APETALA3* (*AP3*) and *PISTILLATA* (*PI*), C- functional genes include *AGAMOUS* (*AG*), and E- functional genes include *SEPALLATA* orthologs (*SEP1*–*SEP4*) [21]. *AP1* plays a crucial role in floral meristem identity and also in sepal and petal development in Arabidopsis [22]. In Arabidopsis, a mutation in *AP1* causes the conversion of sepals into bracts as well as the development of floral buds in the axil of transformed sepals. Moreover, the flowers of the mutant plants also lack petals [23]. Previous studies demonstrated the ability of *AP1*orthologs from various plant species (*J*atropha curcas [24]*,* Orange [25], Pea [26] and Lily [27]) to complement Arabidopsis *ap1* phenotype, which indicates the conservation of the role of *AP1* between different species. However, orthologs of *AP1* have not been yet functionally characterized in rapeseed.

In the current study, we aimed to characterize the function of an *AP1* ortholog in rapeseed using TILLING (Targeting Induced Local Lesions in Genomes). Based on our phenotypic evaluation, we report that a stop codon mutation in *Bna.AP1.A02* alters plant architecture in rapeseed and increases the number of seeds per plant. Moreover, we found that a stop codon mutation in *Bna.AP1.A02* also leads to modifications in floral architecture similar to an Arabidopsis *AP1* mutant phenotype. Our data suggest that EMS-generated alleles can be useful to develop high yielding varieties by conventional breeding and can also be valuable to increase genetic diversity for rapeseed breeding.

## Methods

### Mutation screening

We screened 3840 M_2_ plants of the EMS Express617 winter rapeseed mutant population [28] by TILLING. For TILLING, we used normalized DNA (5 ng/μl) from M_2_ plants which was arranged into ten 96-well microtiter plates using two-dimensional (2D) eight-fold (8x) pooling strategy [28]. We designed paralog-specific primers for *Bna.AP1.A02* and *Bna.AP1.C02* (Additional file 1: Table S1) using the published reference genome sequence [29]. Subsequently, we performed *Cel*I digestion of heteroduplexes, sample purification and polyacrylamide gel electrophoresis (PAGE) on a LI-COR 4300 DNA analyzer (LI-COR Biosciences, http://www.licor.com) according to Harloff et al. [28]. We used GelBuddy Software [30] to identify mutations.

### Plant material and growth conditions

M_4_ seeds were produced by selfing of M_3_ plants of three *Bna.AP1.A02* stop codon mutant families. From each family, 20 M_4_ seeds per genotype (mutant and wildtype) together with Express617 were sown in the greenhouse for phenotyping. All plants were grown in the greenhouse under constant temperature (22 °C) and long day conditions (16 h light, 900 μmol m^− 2^ s^− 1^, Son-T Agro 400 W, Koninklijke Philips Electronics N.V., Eindhoven, Netherlands). After three weeks of pre-culture, we transferred the plants to a cold chamber at 4 °C (vernalization) under long day conditions (16 h light, 200 μmol m-2 s-1, Osram Lumilux T8 L 58 W/840, Osram AG, München, Germany) for eight weeks. After vernalization, we transferred the plants to the initial greenhouse conditions and transplanted them into 11 × 11 cm pots. We randomized the plants two times a week.

### Plant phenotyping and statistical analysis

We measured the total number of seeds per plant, seed yield per plant (g) and a total number of healthy and filled siliques per plant. Moreover, we measured plant height (length of the plant from the base of stem to the top of the main inflorescence at maturity), branch height (distance from the base of the stem to the first branch [1]) and branch number (the total number of primary and secondary branches at maturity). For all the phenotypic traits, we took an average of 15 plants of each, mutant (*aa*), wildtype (*AA*) and Express617. The mean comparison between the genotypes for the investigated traits was performed by ANOVA (Analysis of variance) test (*P* value = 0.0001), while the grouping was done using the LSD test (α ≤ 0.05). For LSD test, we used the R package ‘Agricolae’ version 1.2–8.

### Gene expression analysis

We collected SAM tissue from three M_4_ vernalized plants (BBCH 30) [31] at Zeitgeber time (ZT) 8. We performed RNA isolation from three biological replicates with the peqGold Plant RNA Kit (PEQLAB Biotechnologie GmbH, Erlangen, Germany) according to the manufacturer’s protocol. RNA concentration and purity was determined by agarose gel electrophoresis and photometric quantification with a NanoDrop spectrophotometer (Thermo Scientific). We treated total RNA with DNAse I (Fermentas Inc., Maryland, USA) to remove genomic DNA. Subsequently, we synthesized the first-strand cDNA from 1 μg of DNA-depleted RNA using Oligo (dT)18 primers and the M-MuLV Reverse Transcriptase (ThermoFisher Scientific, Waltham, United States). For quality check, a standard PCR of the synthesized cDNAs (1,10 dilution) was performed with the housekeeping gene *Bna.Actin* (rapeseed actin gene, GenBank Accession No. AF111812). Prior to expression analysis, we developed primers for *Bna.TFL*, *Bna.LFY*, *Bna.SEP4*, *Bna.FUL* and *Bna.AP1* (Additional file 1: Table S1). Moreover, we designed paralog-specific primers for *Bna.TFL* paralogs (*Bna.TFL1.A10*, *Bna.TFL1.C3* and *Bna.TFL1.Ann*) and the amplicons were Sanger sequenced.

We performed quantitative real-time RT-PCR (RT-qPCR) with SYBR qPCR Super mix w/ROX (Invitrogen Corporation, Carlsbad, USA) using a CFX96 Real-Time System (Bio-Rad Laboratories GmbH, München, Germany). For each reaction, we used a total volume of 20 μl containing 100 nM of each primer and 2 μl of diluted cDNA templates with the following cycling conditions: 95 °C for 3 min, 40 cycles of 95 °C for 10 s, 60 °C for 30 s, and 72 °C for 30 s, followed by 95 °C for 10 min. Primer efficiencies for the different targets were determined using four 2-fold serial dilutions of cDNA and were included in the calculation of relative expression levels in Bio-Rad CFX Manager 3.1. We analyzed the amplification curves and used the average Ct values of three technical replicates to calculate relative expression in comparison to the reference gene (*Bna.Actin*) using the ΔΔCt method [32].

## Results

### Identification of *Bna.AP1* stop codon mutants by TILLING

**Fig. 1:**
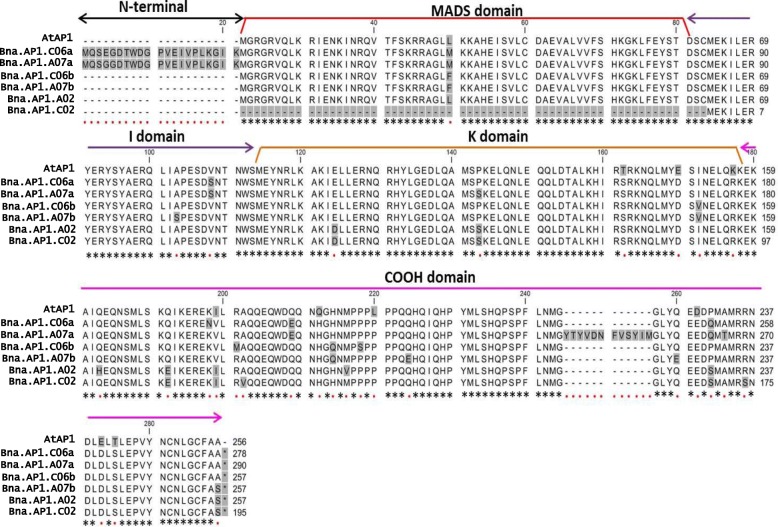
Amino acid alignment between Bna.AP1 proteins and AtAP1 protein. Conserved amino acids between proteins are indicated by asterisks (*), and red color dots (.) indicate non-conserved amino acids. Changes in the amino acids are shaded in grey

**Fig. 2:**
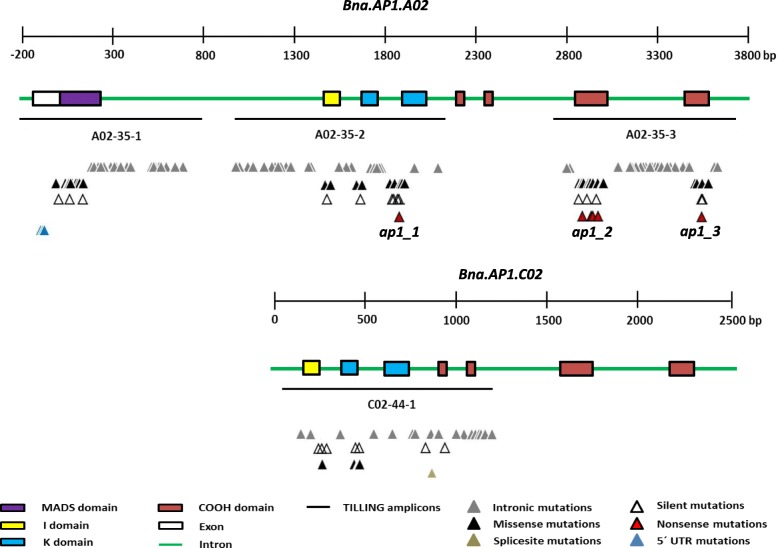
Graphical presentation of EMS-induced mutations in *Bna.AP1.A02* and *Bna.AP1.C02* paralogs in Express617. Only mutations verified by sequencing are shown

We performed amino acid sequence alignments between *A. thaliana* (AtAP1) and six rapeseed (Bna.AP1) AP1 proteins to identify the conserved regions. Based on protein alignments, amino acid sequences are highly conserved between Arabidopsis and rapeseed proteins (Fig. 1). We concluded that all four functional domains present in AtAP1 (MADS domain, I domain, K domain, and COOH domain) are also present in five out of six predicted Bna.AP1 proteins. Only, Bna.AP1.C02 lacks a MADS-box domain based on the published rapeseed genome sequence [29]. We selected two paralogs, *Bna.AP1.A02* and *Bna.AP1.C02* for mutation screening, to study the function of *AP1* orthologs in rapeseed. These two paralogs were selected based on leaf transcriptome analysis data of semi-winter rapeseed cultivar “Ningyou7” [33], expressed sequence tags (EST) data available for winter rapeseed cultivar ‘Darmor-bzh’ in the genome database [29] and the study of genetic variation in *Bna.AP1* paralogs between different *B. napus* morphotypes [34]. Moreover, according to preliminary data from a transcriptome study in shoot apical meristem of rapeseed, *Bna.AP1.A02* and *Bna.AP1.C02* showed higher expression compared to the other four paralogs (Siegbert Melzer, Personal communication). We designed paralog-specific primers and the *Bna.AP1.A02* and *Bna.AP1.C02* amplicons covered 88.7 and 55.9% of the coding sequences, respectively. We screened 3488 M_2_ plants for *Bna.AP1.A02* and 2720 M_2_ plants for *Bna.AP1.C02* paralog to find mutations. After confirmation by sequencing, we found 164 mutations in the *Bna.AP1.A02* paralog and 32 mutations in the *Bna.AP1.C02* paralog (Additional file 2: Table S2). For *Bna.AP1.A02*, we identified six premature stop codon mutations (nonsense) in exon 4, exon 7 and exon 8. Moreover, we also found one splice site mutation in *Bna.AP1.C02* at the 5’end of intron 4 (Fig. 2). We considered three premature stop codon mutants of *Bna.AP1.A02* (annotated as *ap1_1*, *ap1_2,* and *ap1_3*) for further phenotyping in the greenhouse (Additional file 3: Table S3). We selected these three mutants, because the premature stop codon for each of the selected mutant families was on a different exon, resulting in truncated proteins of different lengths. The splice site mutant found in *Bna.AP1.C02* copy was not considered for phenotyping along with three stop codon mutant families, because in an earlier greenhouse experiment, no phenotypic difference was observed between *Bna.AP1.C02* splice site mutant plants and the controls (data not shown).

### Effect of *Bna.AP1.A02* on flowering time, plant architecture and seed yield components

We recorded flowering time, plant height, branch height, number of seeds/plant, seed yield/plant, siliques/plant and branch number/plant of 15 M_4_ plants per genotype for three stop codon mutant families, to investigate the effect of the mutations in *Bna.AP1.A02*. For ease of understanding, the mutant allele was termed ´*a´* and the wildtype allele was termed as ´*A*.´ All three families consisted of mutant (*aa*) and wildtype (*AA*) genotypes, which were obtained by selfing of the M_3_ plants (Additional file 3: Table S3). In this context, a wildtype (*AA*) genotype was generated from the same mutant family and hence, it is expected to have all background mutations as they are in the mutant genotype (*aa*), except at the loci of interest (*Bna.AP1.A02*). Therefore, within each mutant family, we compared the mutant (*aa*) with the wildtype genotype (*AA*). For all the phenotypic traits, the comparison between the mutant (*aa*) and the wildtype genotype (*AA*) was the most important one since they had the same background mutations. This suggests that all the phenotypic variations observed between the mutant (*aa*) and the wildtype genotype (*AA*) plants were due to the stop codon mutation in *Bna.AP1.A02*. We used Express617 as a non-mutated control. We observed that there was no significant difference in the flowering time between mutant and wildtype plants for all three families (data not shown).

**Fig. 3:**
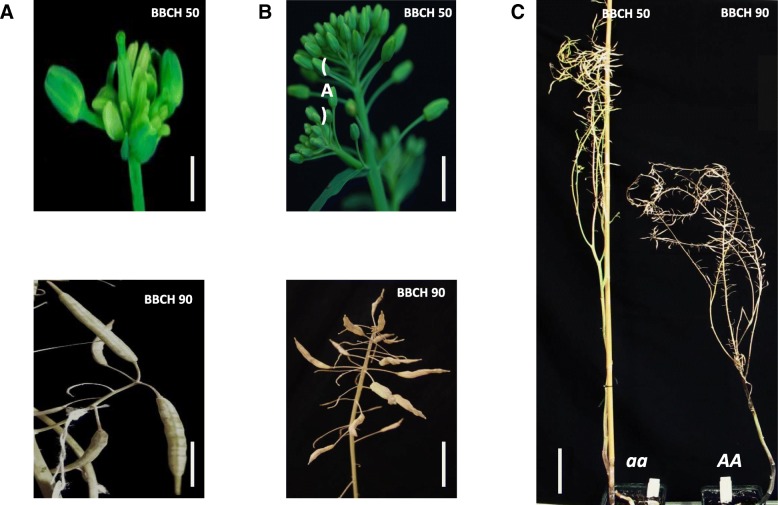
Morphological features of the *ap1_1* stop codon mutant family. Floral buds and silique development in *ap1_1* mutant plants (*aa*) are shown on the left (**a**; scale bar: 1 cm); Floral buds and silique development in homozygous wildtype plants (*AA*) in the center (**b**; scale bar: 1 cm for BBCH50 and 10 cm for BBCH90) and whole plant pictures with both genotypes on the right (**c**; scale bar: 10 cm). Plants were grown in the greenhouse at constant temperature (22 °C), under long day conditions (16 h light) after vernalization (4 °C, 16 h light, 8 weeks)

**Fig. 4:**
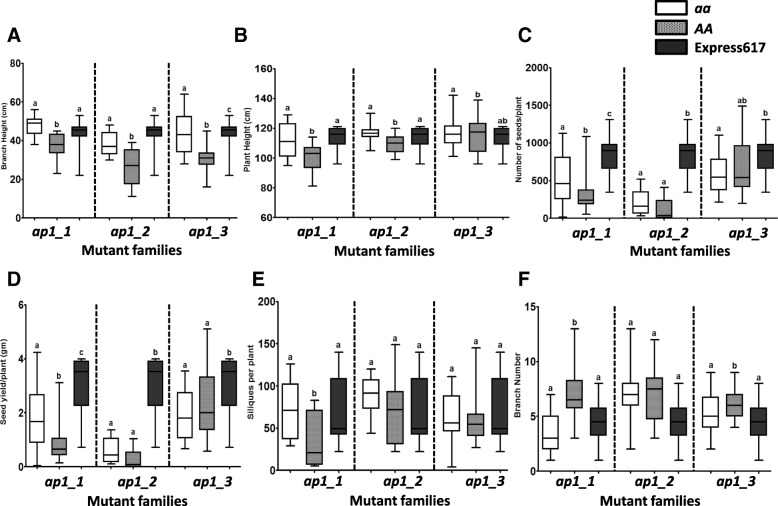
Box plots showing phenotypic analysis of *Bna.AP1.A02* stop codon mutant lines under the greenhouse conditions. **a** Branch height, **b** Plant height, **c** Siliques per plant, **d** Branch number, **e** Number of seeds/plant and **f** Seed yield/plant. 15 plants per genotypes were used for phenotyping. Plants were grown in the greenhouse at a constant temperature (22 °C), and LD (16 h light) after vernalization (4 °C, 16 h light, 8 weeks). Error bars: standard error of the mean for 15 plants. The mean comparison between the genotypes for the investigated traits was performed by ANOVA test (*P* value = 0.0001), while the grouping was done using the LSD test (α ≤ 0.05) in R package ‘Agricolae’ version 1.2–8

The homozygous *ap1_1* mutant plants (*aa*) displayed secondary flower buds instead of sepals, which later developed into siliques (Fig. 3). The same mutant family also exhibited modified plant architecture-related traits like plant height, branch height, and branch number compared to the wildtype plants (*AA*) of the same family (Fig. 4). We observed that plant height and branch height was increased in the mutant plants (116.9 ± 5 cm and 43.0 ± 10.66 cm, respectively) compared to the wildtype plants (109.3 ± 6.3 cm and 31.0 ± 6.8 cm, respectively). Moreover, *ap1_1* mutants demonstrated significant differences in plant yield related traits like seed yield per plant, seed number per plant and siliques per plant compared to the wildtype plants (Fig. 4). *ap1_1* mutant plants had an average seed yield per plant of 1.75 ± 1.1 g compared to 0.84 ± 0.7 g for the wildtype plants.

We observed that *ap1_2* and *ap1_3* mutant plants did not display any variation in floral bud development compared to their respective wildtype genotypes. Nevertheless, *ap1_2* mutant plants exhibited significant differences in plant and branch height, while *ap1_3* mutant plants had significantly different branch height and branch numbers (Fig. 4). We did not observe any significant difference in yield related traits for *ap1_2* and *ap1_3* mutant plants.

### A stop codon mutation in *Bna.AP1.A02* alters the expression of *Bna.TFL1* and *Bna.FUL* in the SAM

**Fig. 5:**
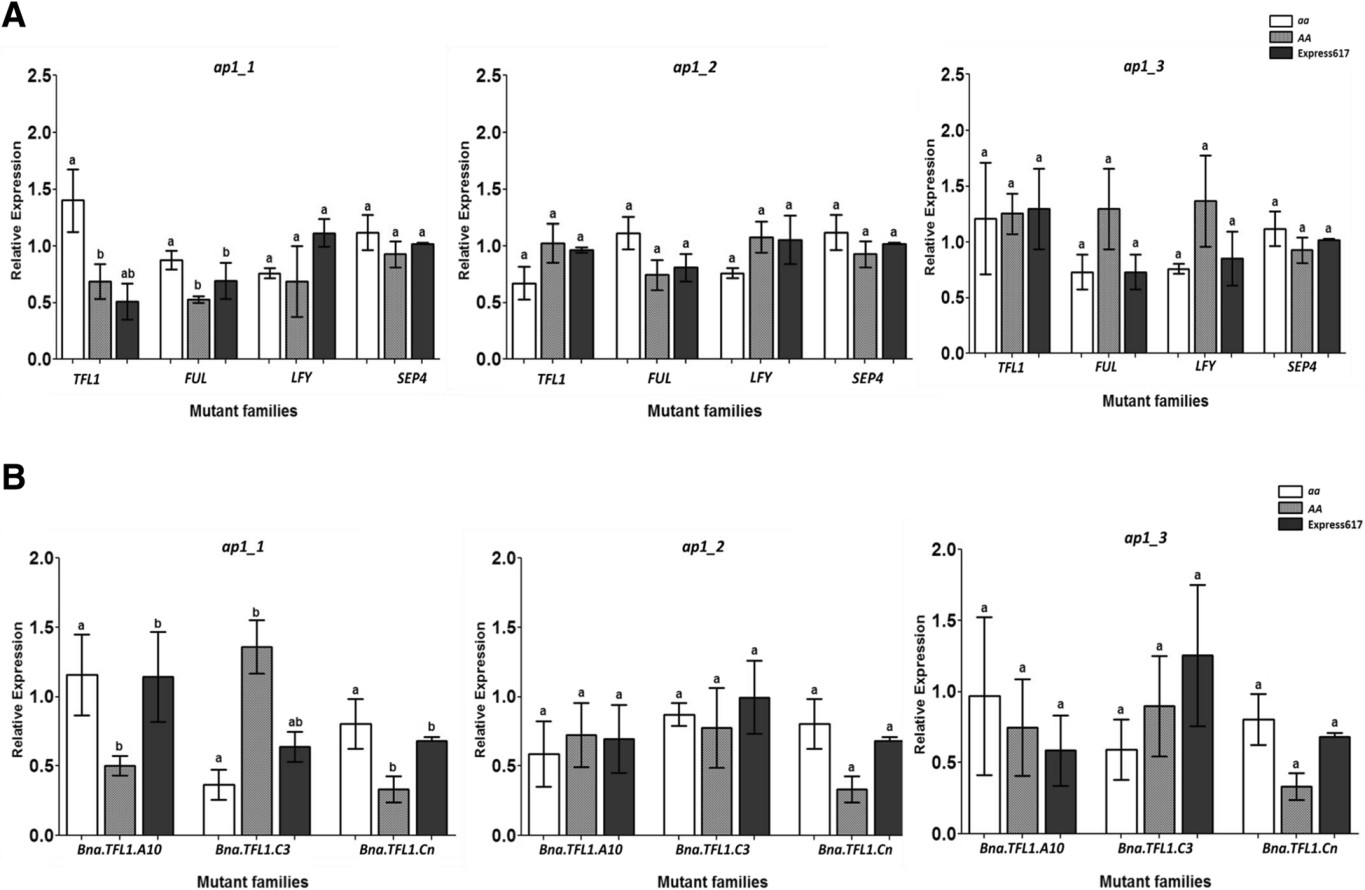
Expression analysis of *Bna.AP1.A02* stop codon mutant lines. **a** Relative expression of *Bna.AP1* putative downstream target genes **b** Relative expression of *Bna.TFL1* paralogs*. aa*: Genotype carrying *Bna.AP1.A02* mutant allele; *AA*: Genotype carrying *Bna.AP1.A02* wildtype allele; Express617: Control. Expression levels of target genes were normalized against *Bna.Actin* total expression. For all genotypes tissue (SAM) sampling was done at BBCH30 between zeitgeber 8 h and 9 h. Three biological replicates and three technical replicates were used for each genotype. Error bars: standard error of the mean for biological replicates. The mean comparison between the genotypes for the investigated traits was performed by ANOVA test (*P* value = 0.0001), while the grouping was done using the LSD test (α ≤ 0.05) in R package ‘Agricolae’ version 1.2–8

We expected that a stop codon mutation in *Bna.AP1.A02* paralog impacts the expression of its downstream target genes. Therefore, we measured the expression of putative downstream target genes in the SAM of *Bna.AP1.A02* stop codon mutant families. We took SAM samples from the greenhouse-grown plants between zeitgeber time 8 and 9. Based on the knowledge from Arabidopsis, we selected *Bna.TFL1*, *Bna.FUL*, *Bna.LFY* and *Bna.SEP4* as putative downstream targets of *Bna.AP1*. We observed ~2-fold higher joined expression of all *Bna.TFL1* paralogs in *ap1_1* mutants compared to wildtype plants (Fig. 5). When we measured the expression of *Bna.TFL1* paralogs separately, we observed a similar expression pattern for three out of four paralogs in *ap1_1* mutants (Fig. 5). Due to high sequence similarity, we could not design paralog-specific primers for *Bna.TFL.Cnn*. We did not observe any significant difference in the expression of *Bna.TFL1* paralogs in *ap1_2* and *ap1_3* mutant plants. To further study the interaction between *Bna.AP1* and *Bna.TFL1*, we reasoned that the expression of *Bna.AP1* also decreases in *Bna.TFL1* mutants, as it is the case for Arabidopsis. For this purpose, we investigated the combined as well as paralog-specific (*Bna.AP1.A02*) expression of *BnAP1* in a *Bna.TFL1.A10* missense mutant family identified in a previous study [35]. We observed that after two generations of backcrossing, the plants carrying a missense mutation in *Bna.TFL1.A10* showed lower combined and paralog-specific (*Bna.AP1.A02*) expression compared to the wildtype plants (Additional file 4: Figure S1).

Besides *Bna.TFL1*, we detected significantly lower expression of *Bna.FUL* in *ap1_1* mutants compared to wildtype, but we did not observe any significant difference in the expression of *Bna.SEP4* and *Bna.LFY* between the mutants and controls. Moreover, we also did not detect any significant difference in the expression of any of the putative downstream target genes in *ap1_2* and *ap1_3* mutant plants (Fig. 5). We observed that there was no significant difference in the *Bna.AP1* combined and paralog-specific (*Bna.AP1.A02*) expression between the mutant and wildtype genotypes for *ap1_1* and *ap1_2* mutant families (Additional file 5: Figure S2). Nevertheless, there was a significant difference in combined and paralog-specific (*Bna.AP1.A02*) expression between the mutant and wildtype genotypes for the *ap1_3* mutant family.

## Discussion

The aim of this study was to characterize the role of *AP1* orthologs in rapeseed. We hypothesized that the function of *AP1* is conserved between rapeseed and Arabidopsis. The main findings of this study can be summarized as follows: (1) a stop codon mutation in *Bna.AP1.A02* strongly affects plant architecture and seed yield-related traits, (2) the same mutation alters the plant architecture and increases the number of seeds per plant. (3) Moreover, the stop codon mutation in the K domain of *Bna.AP1.A02* leads to altered expression of the putative downstream target genes *Bna.TFL* and *Bna.FUL*. Combining all these data, we found compelling evidence that the stop codon mutation in the K domain of *Bna.AP1.A02* leads to architectural changes in rapeseed, with an impact on seed yield components.

TILLING offers a non-transgenic, rapid and cost-efficient method for detection of point mutations, which can be used to target each homologue of a multigene family independently. After phenotypic evaluation of TILLING mutants, favorable alleles can be combined into a single line by crossing single mutant parents. The frequency of EMS mutations depends on several factors like plant species, target tissue, the developmental stage of the target tissue, mutagen and also the concentration of mutagen [28]. Based on the previous studies, mutation frequencies vary in Brassicaceae family. For example, it was reported to be 1/345 kb in Arabidopsis [36], which was lower than mutation frequency in rapeseed (1/41.5 kb, [37]. Moreover, a higher mutation frequency of 1/56 kb was observed in *B. rapa* [38] compared to *B. oleracea*; (1/447 kb, [39]). Apart from Brassicaceae family, Chen et al. [40, 41] reported 1/47 kb mutation frequency in hexaploid wheat, while Till et al. [42] reported 1/300 kb mutation frequency in rice. Based on these studies, it was evident that the mutation frequency in diploid species is expected to be lower than in polyploid species. In our study, we calculated the mutation frequencies of 1/17.4 kb and 1/26 kb for *Bna.AP1.A02* and *Bna.AP1.C02*, respectively. Mutation frequency observed in this study is similar to mutation frequencies reported by Harloff et al. [28] for sinapine biosynthesis genes; (1/12 kb to 1/22 kb) but higher than reported mutation frequencies by Guo et al. [35] for flowering time genes *Bna.TFL1* and *Bna.FT*; (1/24 kb to 1/72 kb), using the same EMS population.

### *Bna.AP1* has functions beyond conferring meristem identity in rapeseed

In the current study, we aimed to evaluate the function of an *AP1* homolog in rapeseed. In Arabidopsis, *AP1* confers meristem identity with an essential role in sepal and petal development [23]. Moreover, an *AP1* mutation in Arabidopsis resulted in delayed flowering [43]. Considering the close phylogenetic relationships between Arabidopsis and rapeseed, we expected that rapeseed plants carrying missense or nonsense mutations in *AP1* paralogs show the same phenotype as Arabidopsis *AP1* mutants. Unlike Arabidopsis, we did not observe any significant difference in flowering time between mutant and wildtype plants, which indicates that *Bna.AP1.A02* does not affect flowering time in rapeseed. However, in a previous study, Schiessl et al. [34] found SNPs in *Bna.AP1* between early and late flowering winter rapeseed lines, suggesting a potential role of *Bna.AP1* in controlling flowering time in rapeseed. One possible explanation for the identical flowering time phenotype of mutant and wildtype plants can be the presence of five non-mutated *Bna.AP1* paralogs in *ap1_1* mutant plants.

We also evaluated floral and plant architecture in *ap1_1* mutant and wildtype plants. We observed the development of floral buds in the axil of transformed sepals in *ap1_1* mutant plants, which confirmed the conserved role of *AP1* as meristem identity gene in rapeseed. However, we did not observe this phenotype in all flowers of the mutant plants. The presence of flowers with normally developed sepals and petals on *ap1_1* mutant plants can be due to the compensation by the other non-mutated paralogs of *Bna.AP1*. A similar phenomenon has been reported for the *INDEHISCENT* (*Bna.IND*) gene, where a mutation in a single paralog of *Bna.IND* gene does not affect the shatter resistance in rapeseed due to the presence of other functional copies of the gene [44]. However, the authors observed a significant increase in shatter resistance, when both paralogs of *Bna.IND* were mutated. Therefore, inducing mutation in all functional copies of *Bna.AP1* might result in a stronger *ap1_1* phenotype.

In polyploid plants like rapeseed, duplicated genes may undergo varying fate like sub−/neo-functionalization [45] or gene loss [46]. We wanted to analyze whether *Bna.AP1.A02* paralog has other functions beyond conferring floral meristem identity in rapeseed. For this purpose, we analyzed the effect of the mutation on plant architecture, yield-related traits as well as the transcriptional activities of its putative downstream target genes. We observed that apart from floral architecture, a stop codon mutation in the K domain of *Bna.AP1.A02* also altered plant architecture-related traits like branch height, plant height and branch number, which is in accordance with studies in other crop plants. In rice (*Oryza sativa* L), plant architecture was altered after overexpression of *OsMADS15*, an ortholog of Arabidopsis *AP1* [18]. Burko et al. [17] showed the role of tomato *AP1*/*FUL* in tomato leaf development. Besides plant architecture, in previous studies, it has been demonstrated that meristem identity genes also affect seed yield-related traits. For instance, in a previous study, it was reported that meristem identity gene, *APETALA2* (*AP2*) controls seed yield and seed mass in Arabidopsis [47]. Furthermore, a homolog of meristem identity gene *FUL* also showed indication of neo-functionalization in rapeseed [33]. Nevertheless, there is no previous report on the role of *AP1* in controlling seed yield related traits in Arabidopsis. Hence, the results from the current study, indicating the involvement of *Bna.AP1.A02* in controlling plant architecture and seed yield related traits hints towards neo-functionalization of the meristem identity gene *AP1* in rapeseed.

### *Bna.AP1.A02* is an upstream regulator of *Bna.TFL1* and *Bna.FUL* in rapeseed meristem

Because *Bna.AP1.A02* stop codon mutation had a substantial effect on plant architecture and yield-related traits; we expected an altered expression of genes which are transcriptionally regulated by *Bna.AP1*. The increased expression of *Bna.TFL1* paralogs in the *ap1–1* mutant in rapeseed is in accordance with the relationship between *TFL1* and *AP1* in Arabidopsis, where constitutive expression of *AP1* downregulated the activity of *TFL1* [48]. A higher expression of *Bna.TFL1* might be the reason for the increased number of branches, and plant height in *ap1_1* mutant plants compared to wildtype plants since previous studies in Arabidopsis and other species have shown that the overexpression of *TFL1* and its homologs causes highly branched inflorescences and thus, altered plant architecture [49–52]. However, the decreased expression of *Bna.AP1* in *Bna.TFL1* missense mutant plants hints towards a different transcriptional regulation between *Bna.AP1* and *Bna.TFL1* in rapeseed. Moreover, the lower expression of *Bna.FUL* in the *ap1_1* mutant in the current study was also in contrast with the expression profile in Arabidopsis, where *FUL* was ectopically expressed in the floral meristem of an *AP1* mutant [53]. Nevertheless, in another study, it was observed that overexpression of Jatropha *AP1* ortholog (*JcAP1*) in Arabidopsis caused higher expression of *FUL* [24]. Hence, the data from current and previous studies suggest that despite of functional conservation, meristem identity genes might display varying transcriptional regulation in other crops compared to Arabidopsis. Based on transcriptional data of *Bna.AP1*, *Bna.FUL* and *Bna.TFL1* from the current study, we propose a model of gene interaction: (1) *Bna.AP1.A02* suppresses the expression of three *Bna.TFL1* paralogs (2) *Bna.TFL1.A10* induces or maintains the expression of *Bna.AP1.A02* and (3) *Bna.AP1.A02* is necessary for maintaining the transcript level of *Bna.FUL*. We propose that analyzing the expression of *Bna.TFL1*, *Bna.FUL* and *Bna.AP1* in CRISPR-Cas mutants of meristem identity genes can depict the transcriptional regulation between these genes in a much clearer way because CRISPR-Cas technology can generate site-specific mutants without any background mutations. The expression and phenotypic data of *Bna.AP1.A02* mutant plants from the current study provide strong evidence for the involvement of this homolog of *AP1* in controlling processes beyond its function as a meristem identity gene in rapeseed. There is evidence of nonsense mediated decay in different crop species like rapeseed [54], wheat [55] and barley [56]. Nevertheless, we did not observe any evidence of nonsense mediated decay in the current study.

Based on phenotypic and expression data, *ap1_1* showed a stronger effect on floral architecture, plant architecture and yield-related traits compared to *ap1_2* and *ap1_3*. This was in accordance with our expectations because *ap1_1* carried a mutation in exon four, which results in the shortest protein among all mutants, lacking a K- and COOH-domain. The other two mutants, *ap1_2* and *ap1_3* carried mutations in exon 7 and exon 8, respectively. Hence, proteins encoded by these mutants are only lacking the COOH domain. There is a considerable amount of evidence in Arabidopsis, emphasizing the significant role of different AP1 domains in the protein function and its interaction with other proteins for the functional specificity. In a previous study [57], chimeric constructs between *AtAP1* and *CAULIFLOWER* (*AtCAL*) were used to demonstrate that K domain and COOH domain of *AP1* are important for conferring floral meristem identity. In another study [58], a 9 bp insertion was found in the exon 4 of *BoAP1-B* gene in *B. oleracea ssp. botrytis* (domesticated cauliflower) and in *B. oleracea ssp. oleracea* (wild cabbage), that lead to a premature stop codon. As a result of these mutations, in both cases, the *BoAP1-B* gene was coding for a truncated protein lacking a part of the K domain and the entire COOH domain, leading to the cauliflower phenotype. Our phylogenetic analysis of AP1 protein sequences from different *Brassicaceae* species revealed that *Bna.AP1.A02* groups strongly with *Bna.AP1.C02* and also a copy from *B. oleracea* (*Bog062650*) (Additional file 6: Figure S3). This provides preliminary evidence about the similar role of these copies in their respective species.

## Conclusions

In the current study, we found that a stop codon mutation in the K domain of *Bna.AP1.A02* leads to altered flower morphology, plant architecture and higher yield compared to the wildtype plants. However, further yield trials in multiple locations and years are needed to investigate the role of *Bna.AP1.A02* in controlling seed yield in rapeseed. Moreover, reduction of the mutation load is required, which can be achieved by backcrossing with an elite line. Besides, marker assisted background selection [59] can be a time-saving approach, where BC_1_ plants are genotyped with numerous markers to select plants with a high share of the recipient genome. This can be achieved with whole genome sequencing data, rapeseed SNP arrays [60] or by AFLP markers (Amplified Fragment Length Polymorphism [61]). Recently, Braatz et al. [62] demonstrated the potential of CRISPR-Cas technology in creating targeted genetic modification in rapeseed. In this way, all paralogs of any gene of interest in rapeseed can be mutagenized at the same time to study the function of the gene. If the effect of the *ap1_1* mutation on yield is proven under field conditions, new *ap1_1* alleles can be selected from the rapeseed gene pool. These alleles and the mutant alleles we created can be introduced into rapeseed breeding programs by conventional backcrossing with elite rapeseed germplasms to produce agronomically superior genotypes.

## Additional files

Additional file 1: **Table S1.** Primers used in this study for screening mutations in Express617 EMS population and for expression analysis by RT-qPCR. (DOCX 19 kb)

Additional file 2: **Table S2.** Details of EMS mutations in *Bna.AP1.A02* and *Bna.AP1.C02* paralogs detected by TILLING of Express 617**.** (DOCX 13 kb)

Additional file 3: **Table S3.** Nucleotide position and amino acid changes in different splice site, non-sense and UTR mutants from *Bna.AP1.A02* and *Bna.AP1.C02* paralogs. The phenotyping was performed with plants from the M4 generation. (DOCX 14 kb)

Additional file 4: **Figure S1.** Relative expression of *Bna.AP1* in BC_2_F_3_ lines homozygous for *Bna.TFL1.A10* missense mutation (Guo et al., 2014). (A) Combined expression of *Bna.AP1* (B) Paralog-specific (*Bna.AP1.A02*). *aa*-M_4_: Genotype carrying *Bna.TFL1.A10* missense mutation (M_4_ generation); *aa*-BC_2_F_3_: Genotype having homozygous *Bna.TFL1.A10* missense mutation in a BC_2_F_3_ generation; *AA*: Genotype carrying *Bna.TFL1.A10* wildtype allele in BC_2_F_3_ generation; Express617: Control. Expression levels of target genes were normalized against *Bna.Actin* total expression. For all genotypes tissue (SAM) sampling was done between zeitgeber 8 h and 9 h. Three biological replicates and three technical replicates were used for each genotype. Error bars: standard error of the mean for biological replicates. The mean comparison between the genotypes for the investigated traits was performed by ANOVA test (*P* value = 0.0001), while the grouping was done using the LSD test (α ≤ 0.05) in R package ‘Agricolae’ version 1.2–8. (PPTX 522 kb)

Additional file 5: **Figure S2.** Relative paralog-specific (*Bna.AP1.A 02*) (Fig. A) and combined expression of *Bna.AP1* (Fig. B) in *Bna.AP1.A02* stop codon mutant lines. *aa*: Genotype carrying *Bna.AP1.A02* mutant allele; *AA*: Genotype carrying *Bna.AP1.A02* wildtype allele; Express617: Control. For all genotypes tissue (SAM) sampling was done between zeitgeber 8 h and 9 h Three biological replicates and three technical replicates were used for each genotype. Error bars: standard error of the mean for biological replicates. The mean comparison between the genotypes for the investigated traits was performed by ANOVA test (P value = 0.0001), while the grouping was done using the LSD test (α ≤ 0.05) in R package ‘Agricolae’ version 1.2–8. (PPTX 428 kb)

Additional file 6: **Figure S3.** Phylogenetic tree of AP1 protein sequences from different *Brassicaceae* species. The tree was constructed using the Neighbour-Joining method. The default bootstrap value was set to 100. The numbers on branches represent bootstrap values in percentage. (PPTX 245 kb)

## Abbreviations

2D: Two-dimensional
8x: Eight-fold
AFLP: Amplified fragment length polymorphism
*AG*: *AGAMOUS*
*AGL*: *AGAMOUS LIKE*
ANOVA: Analysis of variance
*AP1*: *APETALA1*
*AP2*: *APETALA2*
*AP3*: *APETALA3*
*CAL*: *CAULIFLOWER*
*CO*: *CONSTANS*
DH: Doubled haploid
EST: Expressed sequence tags
*FT*: *FLOWERING LOCUS T*
*FUL*: *FRUITFULL*
*IND*: *INDEHISCENT*
IPA: Ideal plant architecture
*LFY*: *LEAFY*
PAGE: Polyacrylamide gel electrophoresis
*PI*: *PISTILLATA*
QTL: Quantitative trait loci
RT-qPCR: Real-time quantitative PCR
SAM: Shoot apical meristem
*SEP*: *SEPALLATA*
*SEP3*: *SEPALLATA3*
*TFL1*: *TERMINAL FLOWER 1*
TILLING: Targeting induced local lesions in genomes
