# Supplementary material for: An APETALA1 ortholog affects plant architecture and seed yield component in oilseed rape (Brassica napus L.)
Source: BMC Plant Biol. 2018 Dec 29;18:380. doi: 10.1186/s12870-018-1606-9 (PMC6310979; doi:10.1186/s12870-018-1606-9)
Supplement: Supplementary file 1 — Table S1. Primers used in this study for screening mutations in Express617 EMS population and for expression analysis by RT-qPCR. (DOCX 19 kb) [file 12870_2018_1606_MOESM1_ESM.docx]

**Additional file 1: Table S1:** Primers used in this study for screening mutations in Express617 EMS population and for expression analysis by RT-qPCR.

| *B.napus* paralog | Primer name | Sequence | Orientation | Amplicon size (bp) | Annealing temperature (^º^C) | Application |
| --- | --- | --- | --- | --- | --- | --- |
| *Bna.AP1.A02* | NE189 | CGTGGACCCATTATTATTACAGTTAAGCC | Forward | 919 | 57 | TILLING |
|  | NE192 | CAGCCAGGAGAACAAAACAATGCATACAG | Reverse |  |  | TILLING |
|  | NE190 | GATCCCGAGACTCACACACAAGTC | Forward | 1323 | 64 | TILLING |
|  | NE194 | CATCTAGCTTAGGGTTCTGAGTGCGG | Reverse |  |  | TILLING |
|  | NE190 | GATCCCGAGACTCACACACAAGTC | Forward | 1002 | 66 | TILLING |
|  | NE193 | CCAGAAGCAGCTGTACGTTTGAAATTG | Reverse |  |  | TILLING |
|  | NE211 | GGACCTCTGGGGATCCACGAAATC | Forward | 1215 | 66 | TILLING |
|  | NE213 | GGTTCTGCAAAGACACACATCACATGATC | Reverse |  |  | TILLING |
|  | NE212 | CAGACT TAGCTCCAGCTGTTCTTCTTCC | Forward | 1153 | 61 | TILLING |
|  | NE213 | GGTTCTGCAAAGACACACATCACATGATC | Reverse |  |  | TILLING |
|  | NE215 | CCATCGCACTACTGCTTGCTT T | Forward | 801 | 60 | TILLING |
|  | NE217 | CACTGGAACTATAACACCTTACTAGC | Reverse |  |  | TILLING |
|  | NE216 | CAAAACGTCGTCTCAACTACCTCT | Forward | 1122 | 66 | TILLING |
|  | NE218 | CCAGTTTCGTCATTCGTTCTCTCCGAA | Reverse |  |  | TILLING |
| *Bna.AP1.C02* | NE203 | GACATGAAGCTAGCCCTAATTCTG T | Forward | 1213 | 66 | TILLING |
|  | NE205 | CAGATGAACAGATCTAGTCGAGGTAGATG | Reverse |  |  | TILLING |
| *Bna.TFL1*- Conserved | SR206 | TGTCTCCTCCAAGCCTAGGGTTG | Forward | 185 | 61 | RT-qPCR |
|  | SR207 | CTCATAGCTCACCACCTCTTTTCC | Reverse |  |  | RT-qPCR |
| *Bna.SEP4*- Conserved | SR215 | GGCATTTGCTAGGTGAAGAGATAGC | Forward | 192 | 63 | RT-qPCR |
|  | SR216 | CCTCCAACTTTCTCTTAAGATCTC | Reverse |  |  | RT-qPCR |
| *Bna.FUL*- Conserved | SR217 | GTAGGGTTCAGCTGAAGAGGATAG | Forward | 156 | 63 | RT-qPCR |
|  | SR218 | CGAAGAGTTTGCCTTTGGAAG | Reverse |  |  | RT-qPCR |
| *Bna.TFL1.A10* | SR175 | GAGCCATTGATAGTGGGAAGAGTGG | Forward | 135 | 62 | RT-qPCR |
|  | SR219 | CTTGGAGGAGACAGCTAAAGGC | Reverse |  |  | RT-qPCR |
| *Bna.TFL1.C03* | SR232 | GCTACAACATGAAGCAAGTCTCCAAC | Forward | 185 | 63 | RT-qPCR |
|  | SR233 | GTTTGTTACTATCCAATGCAGGTGC | Reverse |  |  | RT-qPCR |
| *Bna.TFL1.Ann* | SR234 | CGTGAGTTACAACAAGAAGCAAGTCTG | Forward | 190 | 62 | RT-qPCR |
|  | SR235 | GTTTGTCACAATCCAATGCAGGTG | Reverse |  |  | RT-qPCR |
