# Supplementary material for: An APETALA1 ortholog affects plant architecture and seed yield component in oilseed rape (Brassica napus L.)
Source: BMC Plant Biol. 2018 Dec 29;18:380. doi: 10.1186/s12870-018-1606-9 (PMC6310979; doi:10.1186/s12870-018-1606-9)
Supplement: Supplementary file 2 — Table S2. Details of EMS mutations in Bna.AP1.A02 and Bna.AP1.C02 paralogs detected by TILLING of Express 617. (DOCX 13 kb) [file 12870_2018_1606_MOESM2_ESM.docx]

Additional file 2: Table S2: Details of **EMS mutations in *Bna.AP1.A02* and *Bna.AP1.C02* paralogs detected by TILLING of Express 617**.

|  | ***Bna.AP1.A02*** | ***Bna.AP1.C02*** |
| --- | --- | --- |
| Number of TILLING amplicons | 3 | 1 |
| Coding sequence coverage (%) | 88.7 | 55.9 |
| Number of mutations detected by LICOR gels | 207 | 39 |
| Number of mutations verified by sequencing | 164 | 32 |
| Number of nonsense mutations | 6 | - |
| Number of UTR mutations | 5 | - |
| Number of splice site mutations | - | 1 |
| Number of missense mutations | 40 | 4 |
| Number of silent mutations | 13 | 7 |
| Number of intronic mutations | 100 | 20 |
| Mutation frequency (1/kb)^a^ | 1/17.4^b^ | 1/26^c^ |
| Mutations/1,000 M_1_/1,000 bp | 58 | 38 |

^a^ Mutation frequencies calculated based on the number of mutations/M_1_ plant

^b^ Mutation frequency calculated based on 940 screened M_1_ plants

^c^ Mutation frequency calculated based on 748 screened M_1_ plants
