# Supplementary material for: An APETALA1 ortholog affects plant architecture and seed yield component in oilseed rape (Brassica napus L.)
Source: BMC Plant Biol. 2018 Dec 29;18:380. doi: 10.1186/s12870-018-1606-9 (PMC6310979; doi:10.1186/s12870-018-1606-9)
Supplement: Supplementary file 3 — Table S3. Nucleotide position and amino acid changes in different splice site, non-sense and UTR mutants from Bna.AP1.A02 and Bna.AP1.C02 paralogs. The phenotyping was performed with plants from the M4 generation. (DOCX 14 kb) [file 12870_2018_1606_MOESM3_ESM.docx]

**Additional file 3: Table S3: Nucleotide position and amino acid changes in different splice site, non-sense and UTR mutants from *Bna.AP1.A02* and *Bna.AP1.C02* paralogs**. The phenotyping was performed with plants from the M_4_ generation.

| M_2_ plant | Mutation | Mutant code | Amino acid change | Zygosity of M_2_ | M_3_ seed code | Chi^2^ test for 1:2:1 ratio (*aa: Aa: AA*) | M_4_ seed code |
| --- | --- | --- | --- | --- | --- | --- | --- |
| 48_H3 | *C1865T** | *ap1_1* | Gln126Stop | heterozygous | 160941 | χ² = 4.30 < χ^2^tab= 5.99 ^n.s^ | 160952 (*aa*) |
|  |  |  |  |  |  |  | 160953 (*AA*) |
| 50_A8_c_ | *G2939A** | *ap1_2* | Try186Stop | heterozygous |  | χ² = 1.0 < χ^2^tab = 5.99 ^n.s^ | 160988 (*aa*) |
| 50_B8_c_ |  |  |  |  | 160942 |  | 160989 (*AA*) |
| 58_F1_d_ | *C3486T** | *ap1_3* | Gln227Stop | heterozygous |  | χ² = 0.5 < χ^2^tab = 5.99 ^n.s^ | 160966 (*aa*) |
| 58_G1_d_ |  |  |  |  | 160944 |  | 160970 (*AA*) |
| 42_A1_b_ | C2994T |  | Gln205Stop | heterozygous |  |  |  |
| 42_B1_b_ |  |  |  |  |  |  |  |
| 55_E12 | C2988T |  | Gln203Stop | heterozygous |  |  |  |
| 69_A8_e_ | C3024T |  | Gln215Stop | heterozygous |  |  |  |
| 69_D8_e_ |  |  |  |  |  |  |  |
| 56_D4 | G-1A |  | 5’ UTR | heterozygous |  |  |  |
| 46_H7 | G-29A |  | 5’ UTR | homozygous |  |  |  |
| 61_E12 | G-26A |  | 5’ UTR | heterozygous |  |  |  |
| 56_E10 | G-20A |  | 5’ UTR | heterozygous |  |  |  |
| 69_A8_e_ | G-8A |  | 5’ UTR | homozygous |  |  |  |
| 69_D8_e_ |  |  |  |  |  |  |  |
| 53_G1^f^ | G717A |  | Splice site | homozygous |  |  |  |

^a,b,c,d,e^ : Mutant plants indicated with the same letter belong to the same M_2_ family

^f^ : Mutant family carrying splice site mutation in ***Bna.AP1.C02* paralog**

^n.s^ : Non significant

*: Mutant families used in current study for phenotypic evaluation.
