# Supplementary figures and images for: An APETALA1 ortholog affects plant architecture and seed yield component in oilseed rape (Brassica napus L.)
Source: BMC Plant Biol. 2018 Dec 29;18:380. doi: 10.1186/s12870-018-1606-9 (PMC6310979; doi:10.1186/s12870-018-1606-9)

## Slide 1
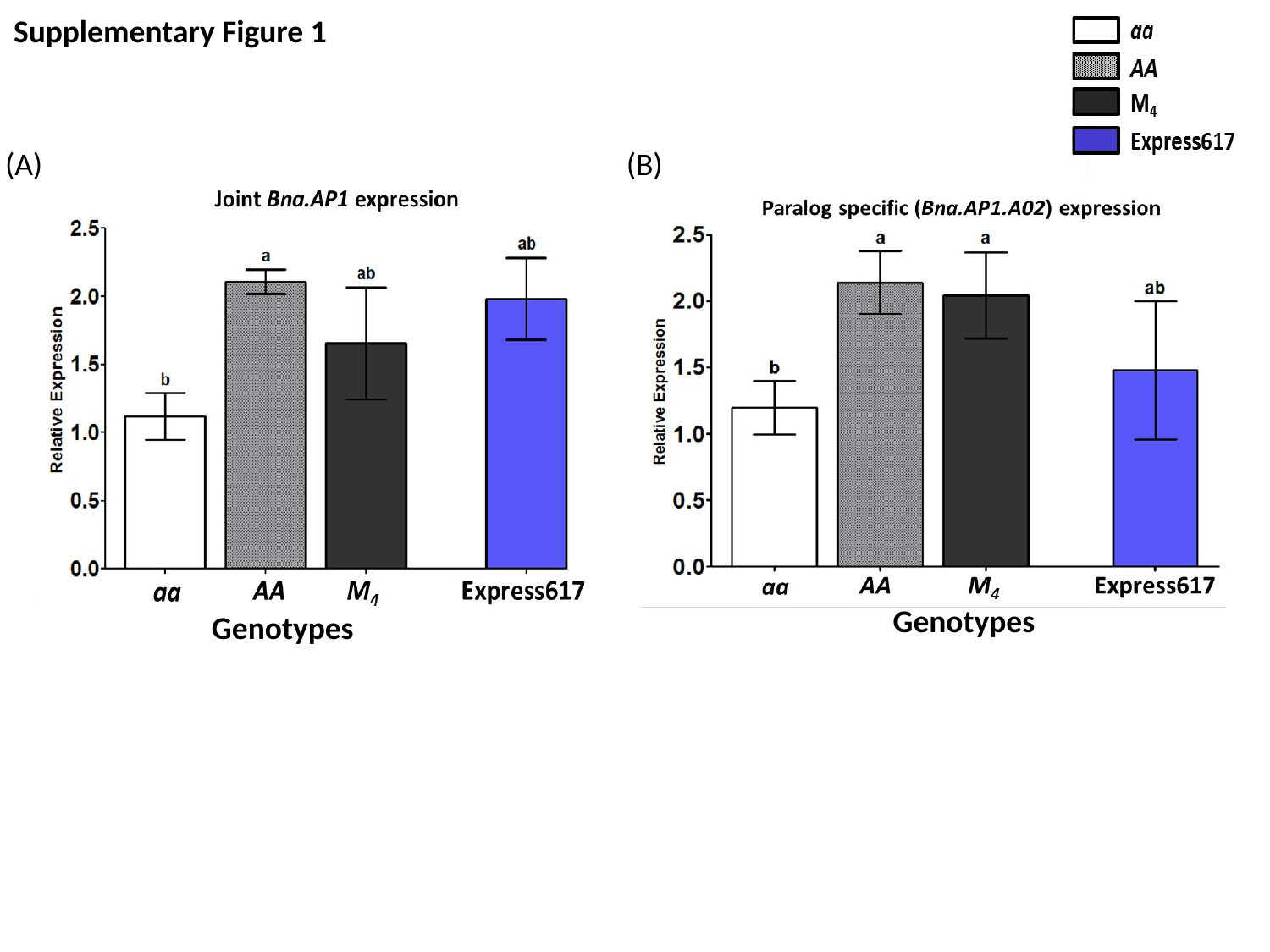

Supplementary Figure 1
(A)
Genotypes
(B)
Genotypes

Supplement: Supplementary file 4 — Figure S1. Relative expression of Bna.AP1 in BC2F3 lines homozygous for Bna.TFL1.A10 missense mutation (Guo et al., 2014). (A) Combined expression of Bna.AP1 (B) Paralog-specific (Bna.AP1.A02). aa-M4: Genotype carrying Bna.TFL1.A10 missense mutation (M4 generation); aa-BC2F3: Genotype having homozygous Bna.TFL1.A10 missense mutation in a BC2F3 generation; AA: Genotype carrying Bna.TFL1.A10 wildtype allele in BC2F3 generation; Express617: Control. Expression levels of target genes were normalized against Bna.Actin total expression. For all genotypes tissue (SAM) sampling was done between zeitgeber 8 h and 9 h. Three biological replicates and three technical replicates were used for each genotype. Error bars: standard error of the mean for biological replicates. The mean comparison between the genotypes for the investigated traits was performed by ANOVA test (P value = 0.0001), while the grouping was done using the LSD test (α ≤ 0.05) in R package ‘Agricolae’ version 1.2–8. (PPTX 522 kb) [file 12870_2018_1606_MOESM4_ESM.pptx]

## Slide 1
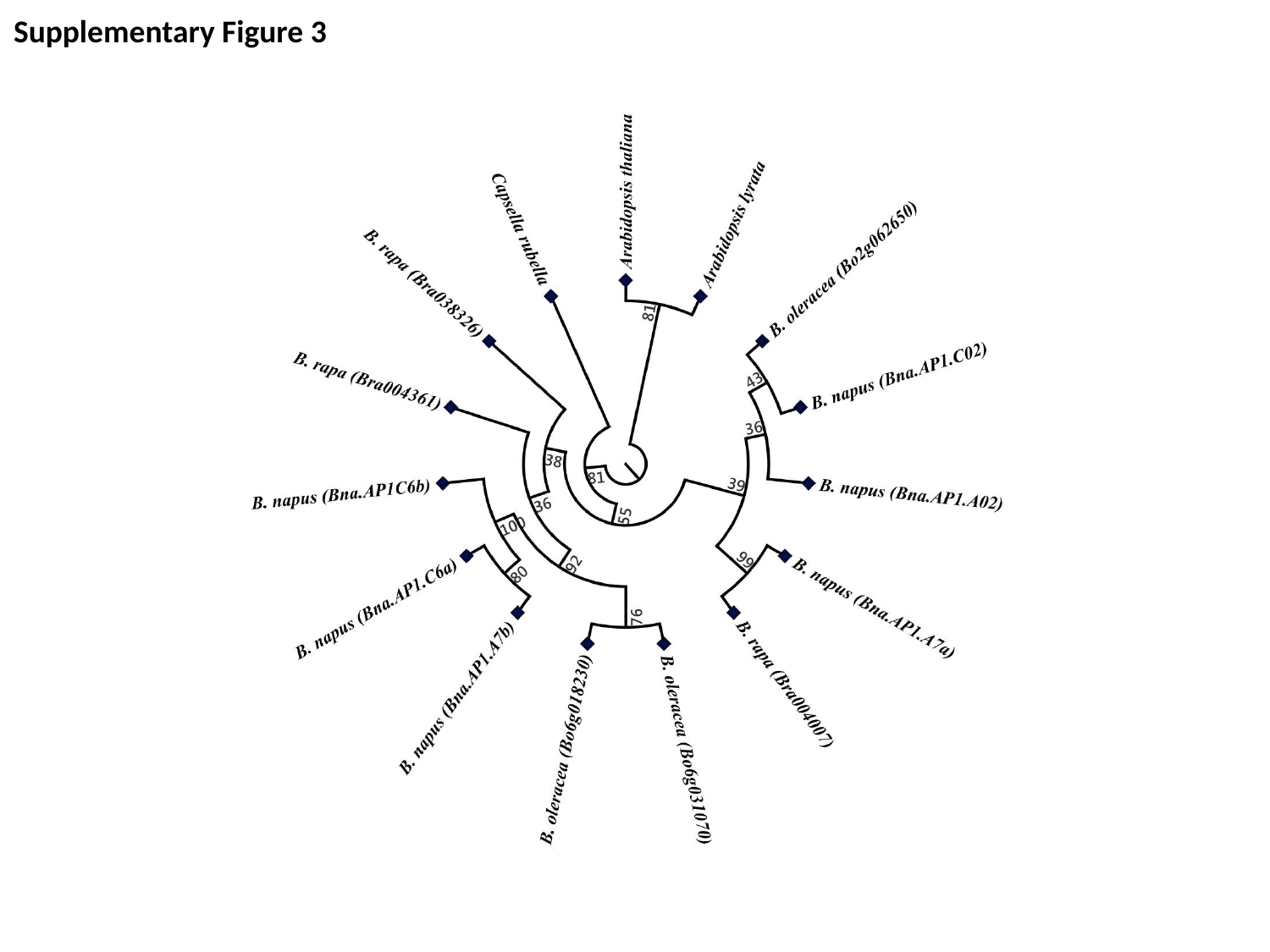

Supplementary Figure 3

Supplement: Supplementary file 6 — Figure S3. Phylogenetic tree of AP1 protein sequences from different Brassicaceae species. The tree was constructed using the Neighbour-Joining method. The default bootstrap value was set to 100. The numbers on branches represent bootstrap values in percentage. (PPTX 245 kb) [file 12870_2018_1606_MOESM6_ESM.pptx]
