# Supplementary material for: An APETALA1 ortholog affects plant architecture and seed yield component in oilseed rape (Brassica napus L.)
Source: BMC Plant Biol. 2018 Dec 29;18:380. doi: 10.1186/s12870-018-1606-9 (PMC6310979; doi:10.1186/s12870-018-1606-9)
Supplement: Supplementary file 5 — Figure S2. Relative paralog-specific (Bna.AP1.A 02) (Fig. A) and combined expression of Bna.AP1 (Fig. B) in Bna.AP1.A02 stop codon mutant lines. aa: Genotype carrying Bna.AP1.A02 mutant allele; AA: Genotype carrying Bna.AP1.A02 wildtype allele; Express617: Control. For all genotypes tissue (SAM) sampling was done between zeitgeber 8 h and 9 h Three biological replicates and three technical replicates were used for each genotype. Error bars: standard error of the mean for biological replicates. The mean comparison between the genotypes for the investigated traits was performed by ANOVA test (P value = 0.0001), while the grouping was done using the LSD test (α ≤ 0.05) in R package ‘Agricolae’ version 1.2–8. (PPTX 428 kb) [file 12870_2018_1606_MOESM5_ESM.pptx]

## Slide 1
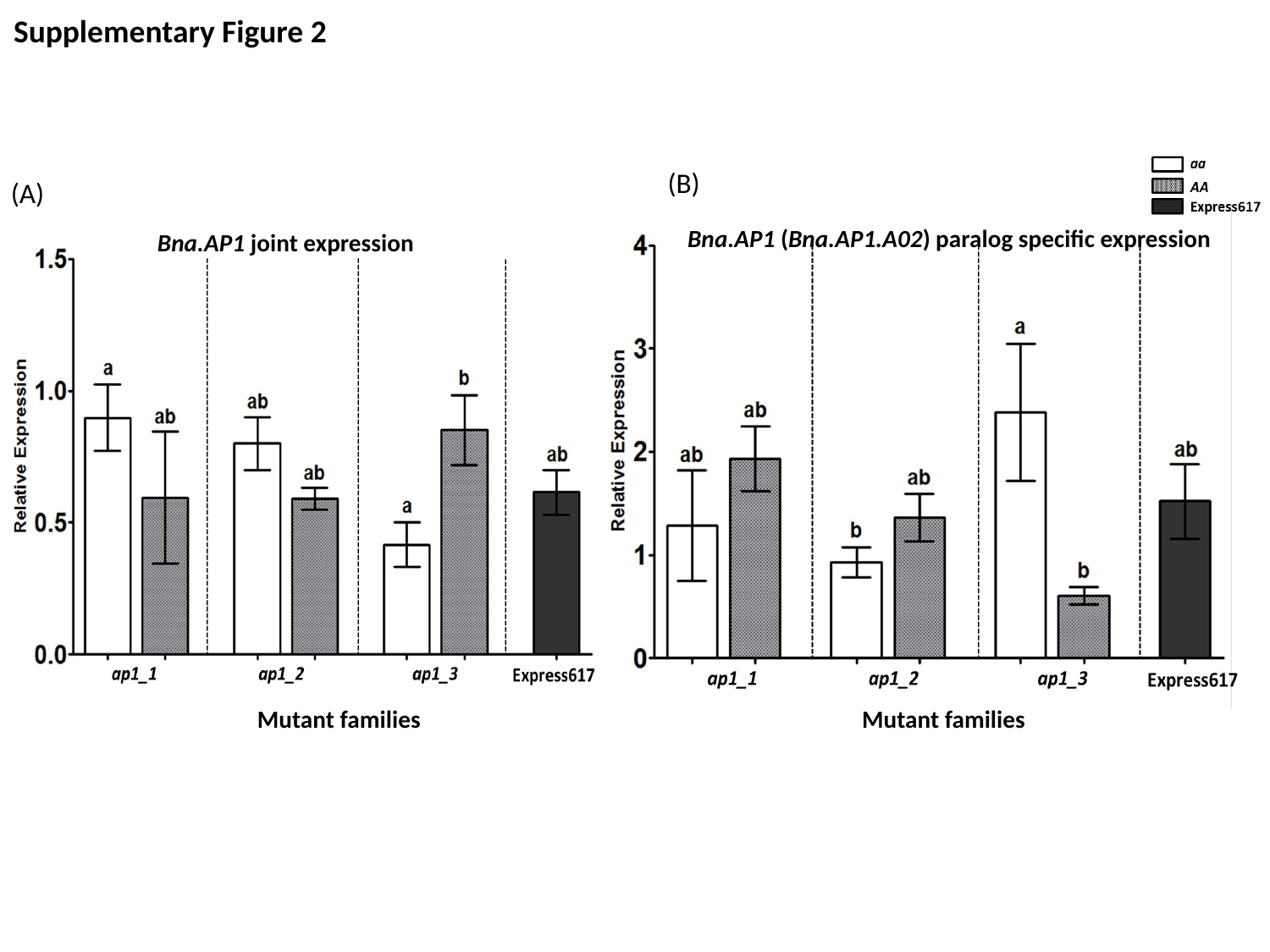

Supplementary Figure 2
(B)
(A)
Bna.AP1 joint expression
Mutant families
Bna.AP1 (Bna.AP1.A02) paralog specific expression
Mutant families
